# Radiosynthesis and first preclinical evaluation of the novel norepinephrine transporter pet-ligand [^11^C]ME@HAPTHI

**DOI:** 10.1186/s13550-015-0113-3

**Published:** 2015-06-10

**Authors:** Christina Rami-Mark, Neydher Berroterán-Infante, Cecile Philippe, Stefanie Foltin, Chrysoula Vraka, Alexander Hoepping, Rupert Lanzenberger, Marcus Hacker, Markus Mitterhauser, Wolfgang Wadsak

**Affiliations:** Department of Biomedical Imaging and Image-guided Therapy, Division of Nuclear Medicine, Medical University of Vienna, Vienna, Austria; Department of Inorganic Chemistry, University of Vienna, Vienna, Austria; Faculty of Life Sciences, Department of Technology and Biopharmaceutics, University of Vienna, Vienna, Austria; ABX Advanced Biochemical Compounds, Radeberg, Germany; Department of Psychiatry and Psychotherapy, Division of Biological Psychiatry, Medical University of Vienna, Vienna, Austria

**Keywords:** NET, PET, Autoradiography, Radiosynthesis, HAPTHI

## Abstract

**Background:**

The norepinephrine transporter (NET) has been demonstrated to be relevant to a multitude of neurological, psychiatric and cardiovascular pathologies. Due to the wide range of possible applications for PET imaging of the NET together with the limitations of currently available radioligands, novel PET tracers for imaging of the cerebral NET with improved pharmacological and pharmacodynamic properties are needed.

**Methods:**

The present study addresses the radiosynthesis and first preclinical evaluation of the novel NET PET tracer [^11^C]Me@HAPTHI by describing its affinity, selectivity, metabolic stability, plasma free fraction, blood–brain barrier (BBB) penetration and binding behaviour in in vitro autoradiography.

**Results:**

[^11^C]Me@HAPTHI was prepared and displayed outstanding affinity and selectivity as well as excellent in vitro metabolic stability, and it is likely to penetrate the BBB. Moreover, selective NET binding in in vitro autoradiography was observed in human brain and rat heart tissue samples.

**Conclusions:**

All preclinical results and radiosynthetic key-parameters indicate that the novel benzothiadiazole dioxide-based PET tracer [^11^C]Me@HAPTHI is a feasible and improved NET radioligand and might prospectively facilitate clinical NET imaging.

**Electronic supplementary material:**

The online version of this article (doi:10.1186/s13550-015-0113-3) contains supplementary material, which is available to authorized users.

## Background

The noradrenergic system—and specifically the presynaptic norepinephrine transporter (NET)—is proposed to be altered in a variety of neurological, neuropsychiatric and cardiovascular diseases. For example, alterations have been shown in Alzheimer’s disease, Morbus Parkinson, major depressive disorder and attention deficit hyperactivity disorder [1–9]. Therefore, a reliable non-invasive molecular imaging technique—such as positron emission tomography (PET)—would be of great benefit for early stage in vivo diagnostics, visualization of treatment response and further elucidation of underlying pathophysiological mechanisms.

Great efforts have been made to develop PET tracers for the NET over the last two decades. Focus was primarily placed on reboxetine-derived ligands [10–14]. However, previous studies have shown that the in vivo and in vitro behaviour of these reboxetine analogues, more specifically [^11^C]MeNER ([^11^C]MRB, ((S,S)-2-(α-(2-[^11^C]methoxyphenoxy)benzyl)morpholine), [^11^C]MeNET and [^18^ F]FMeNER-D_2_ ((S,S)-2-(α-(2-[^18^ F]fluoro[^2^H_2_] methoxyphenoxy)benzyl) morpholine), is not favourable for viable imaging of the NET by PET. Limitations include their metabolic stability, late reaching of equilibrium, unexplainable striatal uptake and complexity of radiosynthesis [10, 15–18]. Recently, we aimed at the preparation of a benzo[d]imidazolone derivative—[^11^C]Me@APPI as new NET PET tracer [19]. Despite its favourable properties and straightforward production, its affinity was not sufficient and its lipophilicity high. Hence, there is ample demand for a novel, improved radioligand for in vivo NET imaging.

**Fig. 1:**
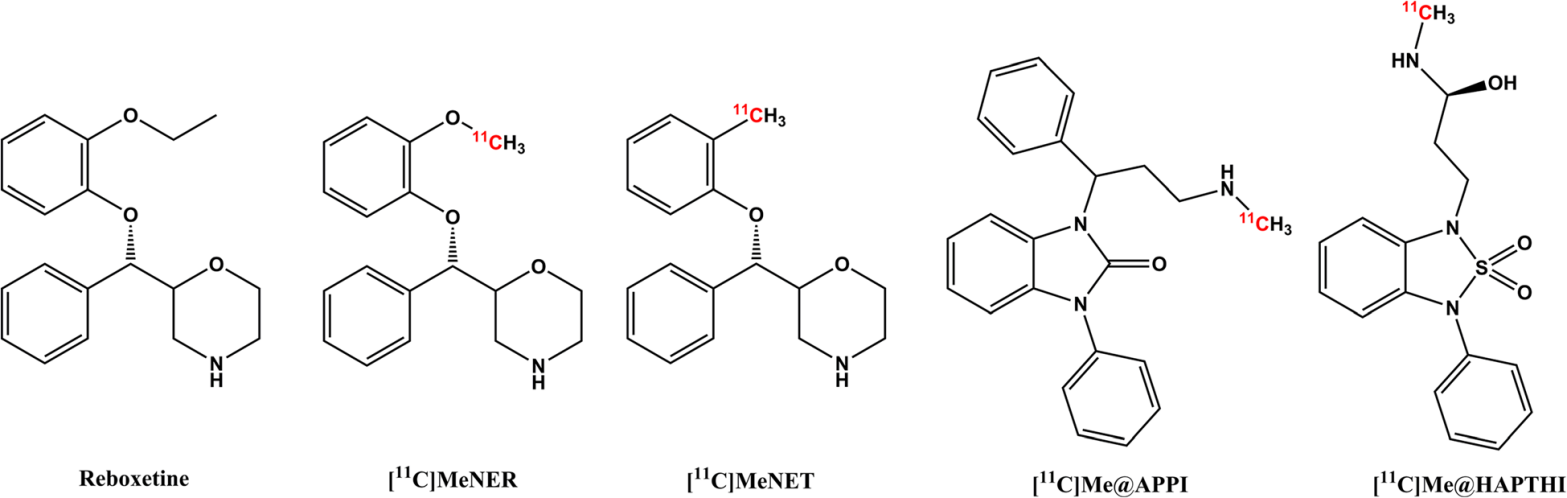
Chemical structures of reboxetine and ^11^C-labelled NET PET tracers [^11^C]Me@APPI and [^11^C]MeNER, [^11^C]MeNET and our novel NET PET ligand [^11^C]ME@HAPTHI. The *red coloured atom* indicates the position of the radioisotope introduced by radiolabeling

Therefore, this study highlights a novel, non-reboxetine-based NET PET tracer based on a benzothiadiazole scaffold: [^11^C]Me@HAPTHI ((S)-1-(3-hydroxy-4-([^11^C]methylamino)butyl)-3-phenyl-1,3-dihydrobenzo[c][1, 2, 5]thiadiazole 2,2-dioxide) (Fig. 1). In general, the designed benzothidiazole dioxides exhibits excellent affinity and selectivity as well as slightly reduced flexibility compared to other previously published benzoimidazolones [20, 21]. Hence, these substances offer an ideal basis for the further development of novel NET ligands for PET imaging.

The objectives of this investigation were as follows:

- The set-up of a small-scale radiosynthetic procedure for the preparation of the carbon-11 labelled [^11^C]Me@HAPTHI and its optimization;
- The up-scaling and set-up of a fully automated preparation of [^11^C]Me@HAPTHI, including purification and formulation;
- The in vitro evaluation of Me@HAPTHI and its precursor HAPTHI. Evaluation includes binding studies for the determination of affinity and selectivity of both Me@HAPTHI and its precursor HAPTHI towards NET using NET, serotonin transporter (SERT) and dopamine transporter (DAT) expressing membranes, metabolic stability testing in vitro against Cytochrom P 450 enzymes, logP analysis and immobilized artificial membrane (IAM) chromatography for indirect measurement of the blood–brain barrier (BBB) penetration and determination of plasma free fraction.
- Comparative in vitro autoradiography on human and rodent tissue slices.

## Methods

### Materials

Precursor, HAPTHI ((S)-1-(4-amino-3-hydroxybutyl)-3-phenyl-1,3-dihydrobenzo[c][1, 2, 5]thiadiazole 2,2-dioxide, and cold reference compound Me@HAPTHI ((S)-1-(3-hydroxy-4-(methylamino)butyl)-3-phenyl-1,3-dihydrobenzo[c][1, 2, 5]thiadiazole 2,2-dioxide) were custom-synthesized by ABX Advanced Biochemical Compounds (Radeberg, Germany). Briefly, synthesis of (2S)-4-(2,2-dioxido-3-phenyl-2,1,3-benzothiadiazol-1(3H)-yl)-1-(methylamino)butan-2-ol followed the route described by Neill et al. [20, 21]. For more details, see Additional file 1.

2-Butanone (MEK, <99.0 % ACS reagent), acetonitrile (ACN, HPLC grade), dimethylsulfoxide (DMSO), tetrabutylammonium hydroxide 30-hydrate (TBAH), ammonium formate, ammonium acetate, sodium hydroxide, triethylamine and ethanol (absolute) were purchased from Sigma-Aldrich (Vienna, Austria) in the highest available grades. In addition, iodine (sublimated grade for analysis; ACS, Pharm. Eur.) was obtained from Merck (Darmstadt, Germany). Silver triflate impregnated carbon was prepared by reaction of 1 g of silver trifluoromethanesulfonate (Sigma Aldrich, Vienna, Austria) in 20 mL ACN with 3 g of Graphpac-GC (80/100 mesh, Alltech, Deerfield, USA). The suspension was stirred under protection from light and in an argon atmosphere for 30 min. After removal of the solvent, the resulting powder was dried under protection from light for further 2 h under reduced pressure.

For formulation of the product, 0.9 % saline solution from B. Braun (Melsungen, Germany), 3 % saline solution (Landesapotheke Salzburg, Austria) and sodium dihydrogenphosphate-monohydrate and disodiumhydrogenphosphate-dihydrate (both from Merck, Darmstadt, Germany) were used. Sterile water was purchased from Meditrade Medicare Medizinprodukte (Kufstein, Austria). Phosphate buffer (125 mM) was prepared by dissolving 0.224 g sodium dihydrogenphosphate-monohydrate and 1.935 g disodiumhydrogenphosphate-dihydrate in 100 mL sterile water. For solid phase extraction, C18 plus SepPak® cartridges were purchased from Waters (Waters® Associates, Milford, USA). Low-protein binding Millex® GS 0.22 μm sterile filters were obtained from Millipore (Bedford, USA).

All other chemicals and solvents for the radiosyntheses were obtained from Merck (Darmstadt, Germany) and Sigma-Aldrich (Vienna, Austria) with at least analytical grade and used without further purification.

NET, DAT and SERT expressing membrane preparations were obtained from Perkin Elmer (MA, USA). An ODP-50 column (20 × 4.0 mm, 5 μm) was purchased from Shodex® (Showa Denko Europe GmbH, Munich, Germany). For prediction of BBB penetration, a Redistech IAM.PC.DD2 column (Regis Technologies Inc., Morton Grove, USA) was used.

Microsomal preparations (human/rat liver microsomes) for stability testing were obtained from BD Bioscience (NJ, USA). Pooled human and rat plasma was obtained from Innovative Research (MI, USA).

The human *postmortem* tissue (7–9 h *postmortem* time, no history of neurological diseases) was obtained from the Neurobiobank of the Medical University of Vienna and approved by the local ethics committee (“Molecular neuropathologic investigation of neurodegenerative diseases” Nr.396/2011) following the principles of the Helsinki Declaration. Wild-type male rats were deeply anesthesized by isoflurane and sacrificed by decapitation. The organs of interest (i.e. brain, heart and testis) were removed and quick-frozen in *i*-pentan. Research using animal tissue was carried out under institutional approval in accordance with the Austrian Animal Care Law. Tissues were cut at −20 °C in a micro-cryotome (Microm HM 560, Thermo Scientific, Austria). Frozen slices were thaw-mounted onto superfrost slides (Menzel-Gläser SUPERFROST plus microscopy slides, Thermo Scientific, Germany). A barrier pen (Mini PAP Pen, Invitrogen, USA) was used for immunohistochemistry only. For detection of autoradiography, a Cyclone Phospho-Imager (Cyclone Plus Storage Phosphor System, Perkin Elmer, Germany) and Phosphor Imager plates (Multisensitive Phosphor Screens Long Type MS, PPN 7001724, Perkin Elmer, Germany) were used. The lead shielded and light-protected cassettes (Fisher Biotech Autoradiography Cassette FBCS 1417) were purchased from Fisher Scientific (PA, USA).

The NET-antibody (SLC6A2 Antibody H-67, sc-67216) was purchased from Santa Cruz Biotechnology (TX, USA). An endogenous Avidin-Biotin blocking kit (ab64212) as well as the DAB (=3,3′-diaminobenzidine) substrate kit (94665) was obtained from abcam (Cambridge, UK). A rabbit primary antibody isotype control was purchased from Invitrogen (CA, USA). A peroxidase-based Vectastain ABC kit (Rabbit IgG, PK-4001) was obtained from Vector Laboratories (CA, USA). Phosphate buffered saline (PBS pH 7.4, tenfold concentrate, 11237) was obtained from Morphisto Evolutionsforschung und Anwendung GmbH (Germany). Mayer’s Hemalaun solution was purchased from Merck Millipore (Germany). Histofluid (Marienfeld Superior, Germany) was used as a mounting medium. Coverslips from Menzel Gläser (24 × 60 mm, Thermo Fisher Scientific, Germany) were used for conservation of mounted slides. All other chemicals were obtained from Sigma-Aldrich.

### Instrumentation

[^11^C]CO_2_ was produced within a GE PETtrace cyclotron (General Electric Medical System, Uppsala, Sweden) by a ^14^ N(p,α)^11^C nuclear reaction under irradiation of a gas target filled with N_2_ (+1 % O_2_) (Air Liquide Austria GmbH, Schwechat, Austria).

The evaluation of the reaction conditions was performed manually with starting activities <2 GBq. After optimization of the reaction parameters, [^11^C]Me@HAPTHI-synthesis was transferred to the TRACERlab™ FX C Pro synthesizer and a fully automated synthesis was established.

Crude [^11^C]Me@HAPTHI was purified by semi-preparative reversed phase HPLC using the built-in semi-preparative HPLC system equipped with a radioactivity and a UV detector (Linear Instruments Model 200 Detector UV/VIS) and a LaPrep HPLC pump (VWR International, Radnor, USA). A Supelcosil^TM^ LC-ABZb, 5 μm, 250 × 10 mm (Supelco®, Bellefonte, PA, USA) column was used with a mobile phase of ACN/0.1 M ammonium acetate 40/60 *v*/*v*% at a flow rate of 6 mL/min.

The analytical HPLC was performed on a Merck-Hitachi LaChrom HPLC system (L-7100 pump; LaChrom L-7400 UV detector) using a NaI radio-detector (Bertholdt Technologies, Bad Wildbach, Germany) and a GinaStar® processing software (Raytest, Straubenhardt, Germany). A Phenomenex® Prodigy, Phenyl-3(PH-3), 5 μm, 250 × 4.6 mm (Phenomenex®, Aschaffenburg, Germany) column with a mobile phase consisting of ACN/0.1 M ammonium formate 50/50 *v*/*v*% at a flow rate of 2 mL/min was used while detection of the cold compounds was performed at 280 nm.

The osmolality of the final sterile product was measured with a Wescor osmometer Vapro® 5600 (Sanova Medical Systems, Vienna, Austria).

## Methods

### Radiochemistry

#### *Production of* [^*11*^*C*]*CH*_*3*_*I and* [^*11*^*C*]*CH*_*3*_*OTf*

The cyclotron production of [^11^C]CO_2_ was terminated at desired target activities between 40 and 50 GBq at currents between 48 and 53 μA (20–25 min) and trapped upon delivery on a molecular sieve (4 Å) within the Tracerlab FxC Pro synthesizer. Subsequently, [^11^C]CO_2_ was converted into [^11^C]CH_4_ by a Ni-catalysed reduction with H_2_ at 400 °C. [^11^C]CH_3_I was produced within the same synthesizer using the dry method (gas phase conversion) described by Larsen et al. [22] with adopted modifications described by Kniess et al. [23]. Briefly, the resulting [^11^C]CH_4_ was reacted with sublimated iodine at 738 °C in a recirculating process for 4 min to give [^11^C]CH_3_I. The produced [^11^C]CH_3_I was trapped on-line on a Porapak® N column and finally released by heating the trap to 190 °C. [^11^C]CH_3_OTf was prepared on-line at the passage of [^11^C]CH_3_I through a pre-heated (200 °C) column containing 300 mg silver triflate impregnated graphitized carbon at a flow rate of 40 mL/min [24].

#### Small-scale reactions

For optimization of reaction conditions, small-scale reactions using [^11^C]CH_3_I or [^11^C]CH_3_OTf were performed. Either [^11^C]CH_3_I or [^11^C]CH_3_OTf was trapped in 500 μL of the solvent of choice at room temperature (RT) and aportioned for further experiments in 1 mL Wheaton v-vials. All evaluation reactions were performed manually (shielded hood; starting activity <2 GBq). The influence of various reaction conditions was investigated:

- Reaction temperature: 25 °C, 75 °C
- Base as catalyst: NaOH, triethylamine (TEA) and TBAH
- Precursor concentration: 1 or 2 mg/mL
- Solvent: MEK or DMSO

**Fig. 2:**
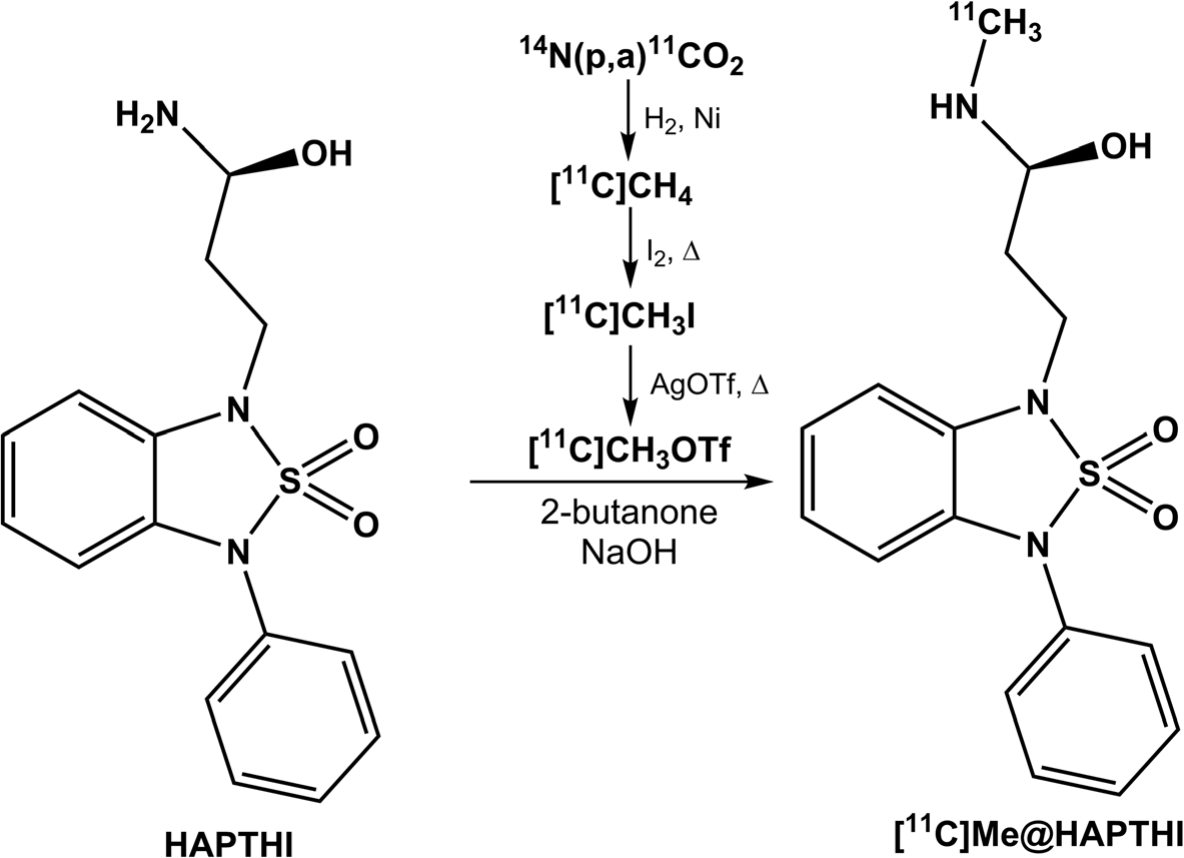
Radiosynthesis of [^11^C]Me@HAPTHI starting from the precursor molecule HAPTHI

Finale reaction volumes of small-scale reactions were 10–200 μL. The reactions were quenched with an equivolume solution of ammonium acetate (aq., pH 3.5), and the radiochemical yield (RCY) was determined using analytical radio-HPLC. In Fig. 2, the reaction scheme is presented.

#### Full automation of radiosyntheses

**Fig. 3:**
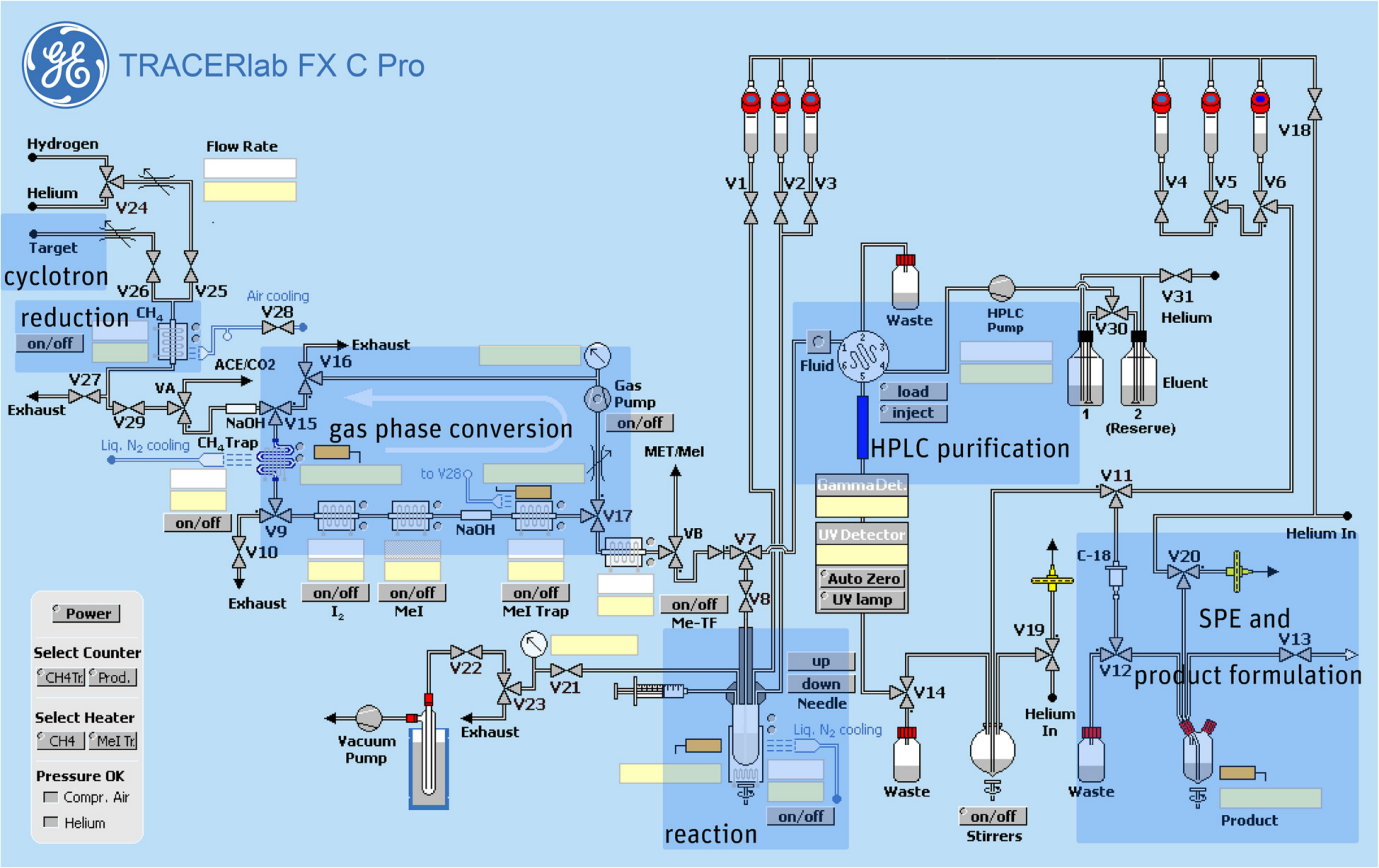
Flow scheme of the fully automated radiosynthesis of [^11^C]Me@HAPTHI

The automation of the *N*-^11^C-methylation reaction was done on the TRACERlab^TM^ FX C Pro (GE Healthcare). A schematic flowchart of the synthesis is depicted in Fig. 3.

**Fig. 4:**
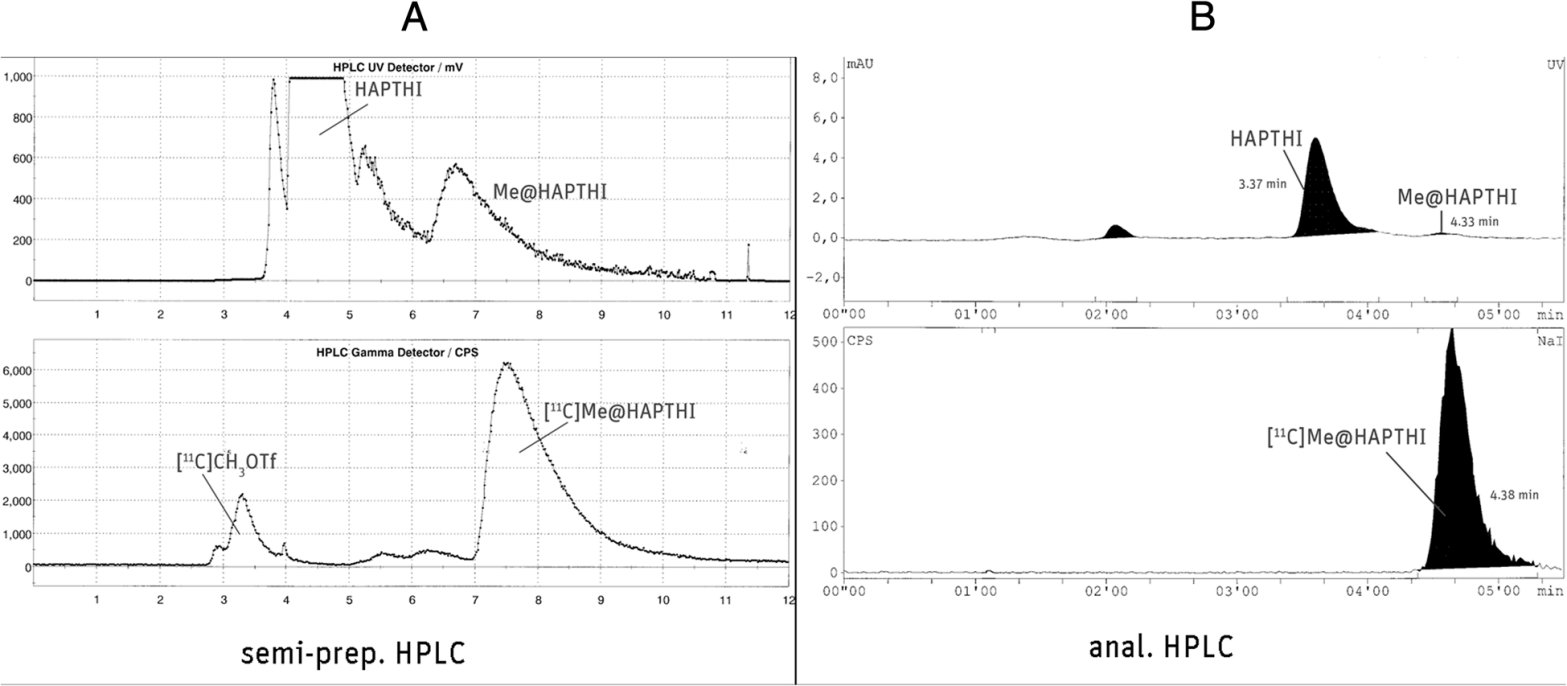
**a** Semi-preparative and **b** analytical HPLC chromatogram

After conversion of cyclotron-produced [^11^C]CO_2_ to [^11^C]methane, [^11^C]methyl iodide and [^11^C]CH_3_OTf, it was trapped at RT in a glass reactor containing precursor HAPTHI (1 mg, 3 μmol) and 0.5 μL of an aqueous NaOH-solution (5 M) in 500 μL MEK. After heating of the sealed reaction vessel to 75 °C for 2 min, the crude reaction mixture was cooled to 25° and quenched by addition of 1 mL HPLC eluent. The entire volume was then transferred to the 5 mL injection loop. The crude mixture was (fluid detector controlled) injected into the semi-preparative HPLC column (Fig. 4). The pure [^11^C]Me@HAPTHI peak was cut into a round bulb, containing 80 mL of distilled water. The now predominantly aqueous product solution was subjected to solid phase extraction by transferring over a preconditioned (10 mL EtOH, air, 20 mL water) C18 SPE cartridge. After rinsing of the C18 SepPak® with water (V6) for complete removal of residual HPLC solvents, the pure product was eluted with 1.5 mL EtOH (V5) into a two-neck vial and the cartridge and transfer lines rinsed with further 5 mL 0.9 % saline into the same vial. After formulation with 9 mL 0.9 % saline, 1 mL 3 % saline and 1 mL 125 mM phosphate buffer, sterile filtration (0.22 μm) was performed under aseptic conditions (laminar air flow hot cell, class A) to avoid microbial contamination.

#### Quality control

Chemical and radiochemical impurities were assessed using analytical radio- and UV-HPLC according to the monograph in the European Pharmacopoeia [25]. Radiochemical identity and purity were measured via analytical radio-HPLC by comparison of retention times with authentic samples. Specific radioactivity was determined by quantification of the non-radioactive product (HPLC UV channel at 280 nm) and inclusion of the overall radiochemical yield (GBq at end of synthesis). Sterility, absence of endotoxins, pH, osmolality and residual solvents were determined by standard procedures routinely performed at the PET Centre of the Vienna General Hospital/Medical University of Vienna and follow the respective monograph in the European Pharmacopoeia [25].

#### Statistical analysis

All quantitative data described in the text and figures are specified as arithmetic mean ± standard deviation. For the determination of significance, a Student’s two-tailed *t* test (*α* = 0.95) was performed using Microsoft® Excel. *P* values of <0.05 were considered to be significant. Unless otherwise stated, error bars in figures represent the standard deviation; if not visual, they are within the icon margin.

### NET-expressing membrane binding studies

The affinity of new radiolabelled ligand was determined in a NET-expressing membrane binding protocol [26, 27]. For details, see Additional file 1.

Data from the competition plots (as arithmetic means of values derived from three different assays, each in triplicate for each compound) were analyzed and subsequently IC_50_ and *K*_i_ values were calculated using GraphPad Prism® software (San Diego, USA).

Assays similar to those described for NET were performed in order to determine the selectivity of the tested compounds towards NET in comparison to DAT and SERT. IC_50_ and *K*_i_ values were obtained in analogy to NET experiments. Ratios DAT/NET and SERT/NET were determined.

### LogD analysis, IAM chromatography and blood–brain barrier penetration

LogD values were assessed using a HPLC-based protocol according to Donovan and Pescatore [28]. All compounds (as cold reference standards) were injected together with two known compounds—with known logD and *k*′ values—according to a standard protocol. A polymeric ODP-50 column was used; a linear gradient from 10 % MeOH 90 % 25 mM phosphate buffer (pH 7.4) to 100 % methanol within 9.4 min at a flow-rate of 1.5 mL/min was applied. Internal standards were triphenylene and toluene; detection was performed at 260 and 285 nm.

As lipophilicity alone was shown to be a tenuous predictor for blood–brain barrier penetration, other in vitro methods have been described, such as immobilized artificial membrane (IAM) chromatography and further calculation of total polar surface area (tPSA) values [29–31]. Therefore, IAM chromatography was performed using a Redistech IAM.PC.DD2 column (15 cm × 4.6 mm) according to previously published methods [19, 32–35]. For analysis, 0.01 M phosphate buffer (pH 7.4) and ACN (in different ratios) were used isocratically as mobile phase at a flow rate of 1 mL/min. Resulting *K*_m_ (membrane partition coefficient) and *P*_m_ (permeability) values were obtained after data analysis using Microsoft Excel. The resulting data were compared with those derived from compounds known to penetrate BBB as external standard. Additionally, tPSA values were determined in silico using Chem Bio Draw Ultra (Cambridge Software, Perkin Elmer, USA).

### Metabolic stability testing

Pooled human and rat liver microsomes are subcellular fractions that are rich in endoplasmatic reticuli, which contain many drug-metabolizing enzymes, e.g. cytochrome P450s, flavin monooxygenases and epoxide hydrolase. Microsomal incubations were performed in order to investigate the metabolization of [^11^C]Me@HAPTHI. As the results, both the percentage of test compound metabolized after a certain time and the biological half-life were determined.

### Plasma protein binding

For the determination of free fraction in human pooled plasma, an ultrafiltration protocol according to previously published methods was used [35–38]. Briefly, aliquots of pooled human plasma were spiked with [^11^C]Me@HAPTHI and centrifuged using ultrafiltration vials (Amicon Centrifree; Millipore, Bedford, USA). The plasma free fraction was calculated, and the percentage of unspecific binding of [^11^C]Me@HAPTHI to the filter matrix evaluated. For a detailed method, see Additional file 1.

### Autoradiography, Nissl staining and immunohistochemistry

Human brain tissue (cortex, thalamus, hippocampus, cerebellum and hypothalamus) was obtained deeply frozen from the Neurobiobank of the Medical University Vienna and was stored at −80 °C. Before cutting, tissue blocks were thawed slowly within 12 h to −20 °C. The organs were cut at −20 °C in a micro-cryotome into 10-μm-thick slices and thaw mounted onto object slides. Slices were again stored at −80 °C until the beginning of the experiment.

In vitro autoradiography was performed with slight modifications according to previously published protocols [13, 39, 40]. Non-specific binding was determined by co-incubation with excess Nisoxetine (10 μM). For competition experiments, non-radioactive FMeNER-D2, an established NET PET tracer, and Me@HAPTHI were added to the incubation solution in different concentrations. After 1 h at room temperature, incubation was stopped and slices were processed on phosphor imaging films.

All data was exported to Microsoft Excel for statistical analysis, and the percentage of total specific binding was calculated.

Post-autoradiographic processing of the slices was done by Nissl staining in order to facilitate morphological mapping of hot areas in the autoradiography. The same tissue slices were stained after autoradiography with cresyl violet [28, 41, 42] to demonstrate the Nissl substance in the neurons and cell nuclei. For a detailed procedure, see Additional file 1.

Immunohistochemical staining experiments were performed on rat and human tissue cryo-slices, vicinal to the slices used for autoradiographic experiments. The staining procedure was a modification of a general protocol as published previously in detail [28, 43].

## Results

### Radiochemistry

**Fig. 5:**
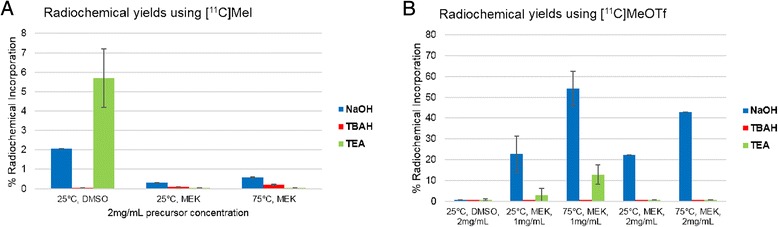
Dependence of the radiochemical yield of [^11^C]Me@HAPTHI (*n* ≥ 3) on the ^11^C-methylation agent **a** [^11^C]methyliodide or **b** [^11^C]methyltriflate) in DMSO and 2-butanone using different bases (NaOH, triethylamine or TBAH) at 2-min reaction time

The optimum parameters were examined in small-scale reactions. Thus, the influence of different ^11^C-methylation agents, solvent, precursor concentration, reaction temperature and base were investigated (Fig. 5a–d). Radiochemical yields (RCY) of [^11^C]Me@HATPHI were below 6 % for all examined conditions using [^11^C]CH_3_I as methylation agent. Hereby, DMSO proved to be the best solvent for the SN_2_ reaction using [^11^C]methyl iodide. In contrast, very promising results were obtained using [^11^C]CH_3_OTf as radio-methylation agent (Fig. 5c–d). Interestingly, the use of DMSO as solvent did not result in high yields, less than 1 % RCY was observed using [^11^C]CH_3_OTf. Applying 2-butanone resulted in high radiochemical yields. Furthermore, the influence of basic catalysis was examined: TBAH catalysis could not shift the reaction kinetics to favourable outcomes, as it did not result in any methylation of HAPTHI. Up to 12.8 ± 4.7 % RCY was observed when using 0.5 μL triethylamine instead. Conducting the experiments with 0.5 μL of 1 M NaOH (aq.), however, yielded 42.9 ± 5.2 % radiochemical yield with 1 mg/mL precursor concentration and even above 50 % RCY were obtained with 2 mg/mL precursor concentration. A further increase in basicity—facilitated by 0.5 μL 5 M NaOH (aq.) instead of 1 M NaOH (aq.)—did not lead to improved results (in a total reaction volume of 100 μL); only <0.5 % RCY were obtained.

Hence, the best results were obtained with NaOH-catalysis in 2-butanone for 2 min at 75 °C using 2 mg/mL precursor HAPTHI. Thereby, 54.0 ± 8.3 % radiochemical yield was achieved.

Therefore, these optimum reaction parameters were transferred to the fully automated radiosynthesis within the Tracerlab FxC Pro synthesizer. In Table 1, an overview on the automated syntheses, their conversion and yield is given. The crude reaction mixture was purified via semi-preparative radio-HPLC using isocratic conditions (0.1 M ammonium acetate and acetonitrile (60/40; *v*/*v*)) at a flow rate of 5 mL/min. An exemplary semi-preparative HPLC chromatogram is outlined in Fig. 4a. The precursor HAPTHI was found to be eluted at a retention time of 4.5 min (*k*′ = 0.55) and the product [^11^C]Me@HAPTHI at 7.6 min (*k*′ = 1.62), respectively.

**Table 1:** Overview on the fully automated, large-scale radiosyntheses of [^11^C]Me@HAPTHI

[^11^C]Me@HAPTHI (*n* = 7)	Mean	SD	Median
Starting activity [^11^C]CO2	53.4	2.4	53.9
Trapped [^11^C]CH4	34.6	4.6	32
Trapped [^11^C]CH3I	29.6	2.4	29
Trapped [^11^C]CH3OTf in reactor	16.6	5.5	17.2
After quenching	8.8	3.6	8.9
Loss during injection in loop waste	1.0	0.5	0.8
Product [^11^C]Me@HAPTHI (EOS)	2.2	2.0	1.9
Yield (decay corr. to EOB)	13.7	13.5	15.9
Specific activity [GBq/μmol] (EOS)	43.4	29.7	59.2

Reaction conditions: [^11^C]MeOTf, NaOH, MEK, 2 mg/mL precursor concentration

*EOS* end of synthesis, *EOB* end of bombardment

Overall, seven large-scale radiosyntheses were performed, yielding 2.2 ± 2.0 GBq (18.9 ± 13.3 %, corrected for decay to EOB) of sterile, formulated [^11^C]Me@HAPTHI within 41 min including 5 min of radiopharmaceutical quality control. A mean specific activity of 46.8 ± 28.5 GBq/μmol was found in the large-scale syntheses (calculated using an HPLC-based method). A representative analytical HPLC chromatogram of the purified, sterile [^11^C]Me@HAPTHI is shown in Fig. 4b. The retention times in the analytical HPLC assay were 3.37 min (*k*′ = 2.17) for precursor HAPTHI, 1.8 min (*k*′ = 0.7) for [^11^C]MeOH, 2.7 min (*k*′ = 1.55) for [^11^C]CH_3_OTf and 3.1 min (*k*′ = 1.9) for [^11^C]CH_3_I, respectively. The product [^11^C]Me@HAPTHI was eluted at a retention time of 4.38 min (*k*′ = 3.08). Radiochemical purity always exceeded 98 %. Osmolality and pH values were at all times found to be in a physiological range. Residual solvent analysis using GC revealed MEK <5 ppm and ACN <20 ppm, besides 8.5 % ethanol present in the product formulation (total product volume 17.5 mL). Moreover, sterility and absence of endotoxins was approved for all produced batches of [^11^C]Me@HAPTHI upon complete decay of radioactivity as *in*-*process* control.

### Affinity and selectivity testings

Affinity of reference compounds (Me@HAPTHI and its radiolabeling progenitor HAPTHI) was determined using human NET membranes as *K*_d_ = 0.21 ± 0.07 nM for Me@HAPTHI and 24.2 ± 10.9 nM for HAPTHI, respectively (*n* ≥ 9 triplicates). For determination of selectivity, the affinity of both reference substances was assessed on human DAT and SERT membranes and revealed >10 μM for both compounds for DAT and 409 ± 43 nM (Me@HAPTHI) and 10,274 ± 1207 nM (HAPTHI) towards SERT, respectively, (*n* ≥ 5 triplicates). Hence, selectivity of Me@HAPTHI towards NET was determined as DAT/NET >1947.6 and SERT/NET = 9757. Both values clearly elucidate the ideal binding properties of our novel NET PET ligand [^11^C]Me@HAPTHI.

### LogD analysis, IAM chromatography and blood–brain barrier penetration

The lipophilicity of the novel NET PET radioligand Me@HAPTHI was found to be in a decent range (logD = 2.27 ± 0.01) for a potential penetration of the BBB. The precursor HAPTHI showed a logD value of 2.30 ± 0.01. Additionally, BBB penetration experiments using IAM chromatography revealed a permeability of *P*_m_ = 1.15 ± 0.25 for Me@HAPTHI and *P*_m_ = 1.14 ± 0.27 for the precursor HAPTHI, respectively. Both values were within the identical, ideal range (*P*_m_ = 0.01–4.21) from other PET tracers, known to easily penetrate the BBB [34].

### Metabolic stability testing

**Fig. 6:**
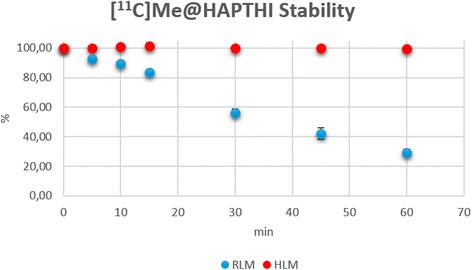
Metabolic stability of [^11^C]Me@HAPTHI against human and rat liver microsomes

Stability testing using human liver microsomes (*n* = 4) revealed no significant metabolism of [^11^C]Me@HAPTHI within the tested timeframe. After 60 min, 99.6 ± 0.3 % of the tracer was found to be still intact. Incubation of [^11^C]Me@HAPTHI with pooled male rat liver microsomes revealed a higher metabolic degradation. The percentage of intact tracer over time is presented in Fig. 6. Overall, 29.3 ± 1.9 % tracer was still intact after 1-h incubation time. Thus, the stability of the novel NET PET tracer [^11^C]Me@HAPTHI is encouraging in a human and rodent setting and superior to the established reboxetine-derived PET tracer [^18^ F]FMeNER-D2.

### Plasma protein binding

The mean percentage of plasma free fraction (ff) and percentage of unspecific binding to the filter matrix of the Centrifuge vials was determined. A plasma free fraction of ff = 8.2 ± 0.3 % (*n* = 7 triplicates) as well as an unspecific filter retention of 51.26 ± 0.78 % was found. Overall, the ff of our novel NET PET tracer [^11^C]Me@HAPTHI was in the same range as that of [^11^C]ADAM [35].

### In vitro autoradiography, immunohistochemistry and Nissl staining

**Fig. 7:**
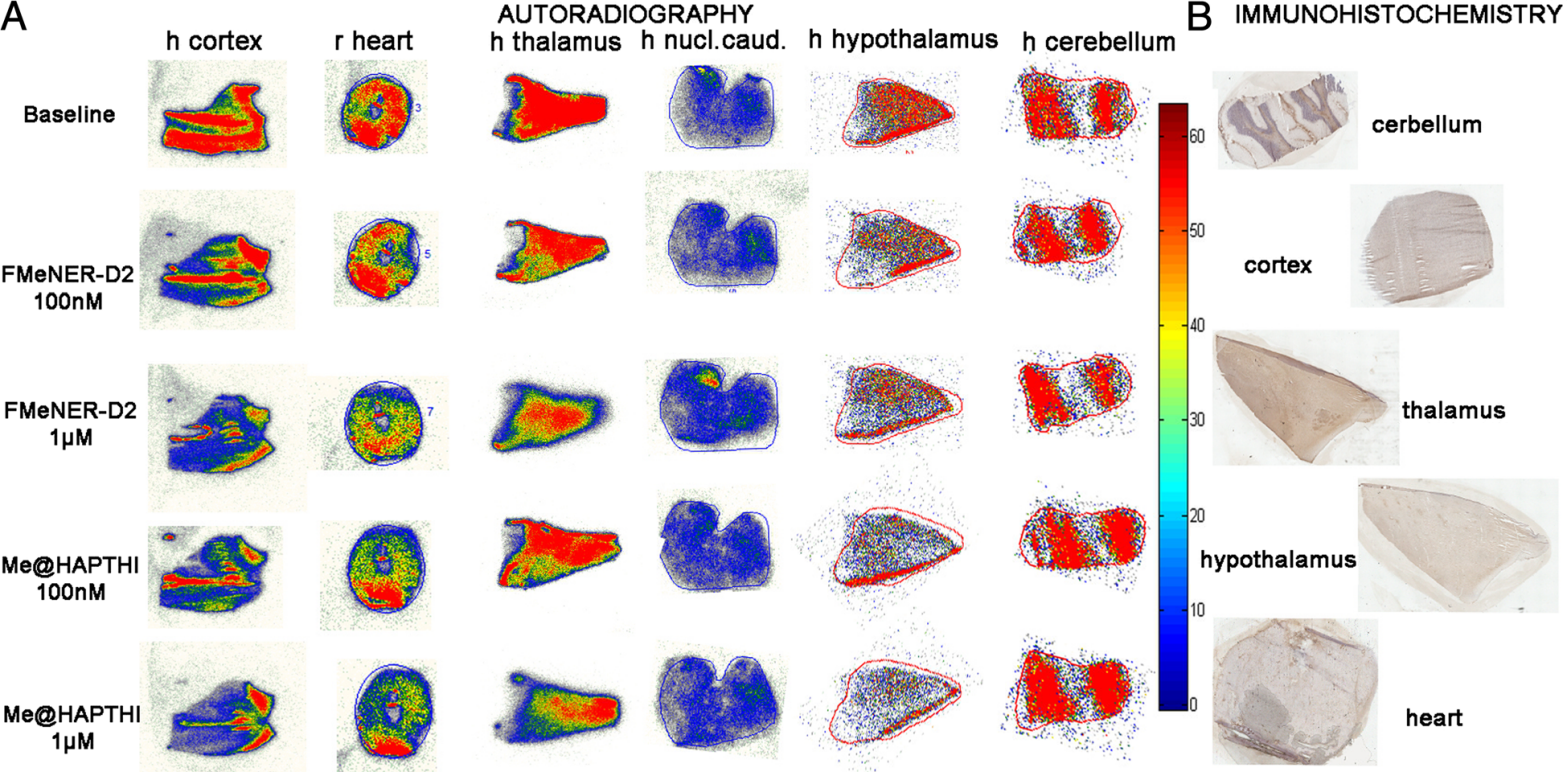
**a** NET-autoradiography and **b** immunohistochemistry of [^11^C]Me@HAPTHI on 10 μm slices of human cortex, thalamus, hypothalamus, cerebellum and nucleus caudatus as well as rat heart tissue and blocking with 100 nM FMeNER-D2, 1 μM FMeNER-D2, 100 nM Me@HAPTHI and 1 μM Me@HAPTHI. The scale shows the radioactivity from high (*red*) to low levels of radiotracer present on the Phosphor imager film

In the autoradiographic experiments, the highest uptake of [^11^C]Me@HAPTHI was observed in NET-rich regions identified with immunohistochemistry (Fig. 7). Blocking was performed with non-radioactive NET ligands FMeNER-D2 and Me@HAPTHI in two different concentrations each (100 nM, 1 μM). A concentration-dependent binding displacement was observed using human tissue samples for both cold competitors. In Table 2, an overview on the percentage of specific displaceable binding of [^11^C]Me@HAPTHI and fmol/mm^2^ values of relative transporter protein density on the different tissue sections is given. All values are given in % as mean *n* ≥ 3 triplicates. Autoradiography of human cerebellum revealed NET specific uptake in NET-rich regions identified with IHC, though blocking experiments were not possible due to the vast inhomogeneity of the tissue samples. In human nucleus caudatus, a region known to be low in NET density, only unspecific binding was observed.

**Table 2:** Overview of specific NET binding of the radioligand [^11^C]Me@HAPTHI vs. Me@HAPTHI and FMeNER-D2 on rat and human tissue origin

*n* ≥ 3	[^11^C]Me@HAPTHI	
	% BL-competitor	fmol
Rat heart		
FMeNER 1 μM	88.8 ± 11.2	<0.01
FMeNER 100nM	99.00 ± 0.07	<0.01
Me@HAPTHI 1 μM	92.5 ± 7.5*	<0.01
Me@HAPTHI 100nM	104.5 ± 4.5	<0.01
Human cortex		
FMeNER 1 μM	71.9 ± 7.9*	0.86
FMeNER 100nM	86.3 ± 11.2*	<0.01
Me@HAPTHI 1 μM	66.3 ± 5.9*	1.32
Me@HAPTHI 100nM	82.1 ± 13.9*	0.02
Human thalamus		
FMeNER 1 μM	68.36 ± 2.11	0.71
FMeNER 100nM	77.6 ± 9.8	0.47
Me@HAPTHI 1 μM	85.9 ± 18.5	0.09
Me@HAPTHI 100nM	92.5 ± 17.3	0.26
Human hypothalamus		
FMeNER 1 μM	77.4 ± 14.5	0.02
FMeNER 100 nM	97.8 ± 14.6	0.11
Me@HAPTHI 1 μM	62.0 ± 3.6*	0.04
Me@HAPTHI 100 nM	83.7 ± 1.7*	0.05
Human hippocampus		
FMeNER 1 μM	67.3 ± 8.2	<0.01
FMeNER 100 nM	97.1 ± 10.3	<0.01
Me@HAPTHI 1 μM	68.3 ± 5.3	<0.01
Me@HAPTHI 100 nM	84.1 ± 9.3	<0.01
Human nucleus caudatus		
FMeNER 1 μM	107.6 ± 17.7	n.d.
FMeNER 100nM	102.6 ± 14.5	n.d.
Me@HAPTHI 1 μM	110.0 ± 21.0	n.d.
Me@HAPTHI 100nM	93.5 ± 12.5	nd
Human cerebellum		
FMeNER 1 μM	108.2 ± 17.3	n.d.
FMeNER 100nM	103.9 ± 12.2	n.d.
Me@HAPTHI 1 μM	107.2 ± 20.8	n.d.
Me@HAPTHI 100nM	124.7 ± 10.8	n.d.

fmol values reflect calculated relative concentration (fmol/mm^2^) of transporter protein). Limit of detection = 0.01 fmol; BL=baseline

*n.d.* not determined

*p < 0.05

Immunohistochemical staining was used to allocate areas with high uptake in autoradiography with regions known high NET abundance. Hence, the NET antibody-dye complexes were found highly abundant in the heart fibres, hippocampus, thalamus and hypothalamus and to a minor extent in all other brain regions (Fig. 7). NET specificity of staining was validated using a rabbit antibody isotype control.

**Fig. 8:**
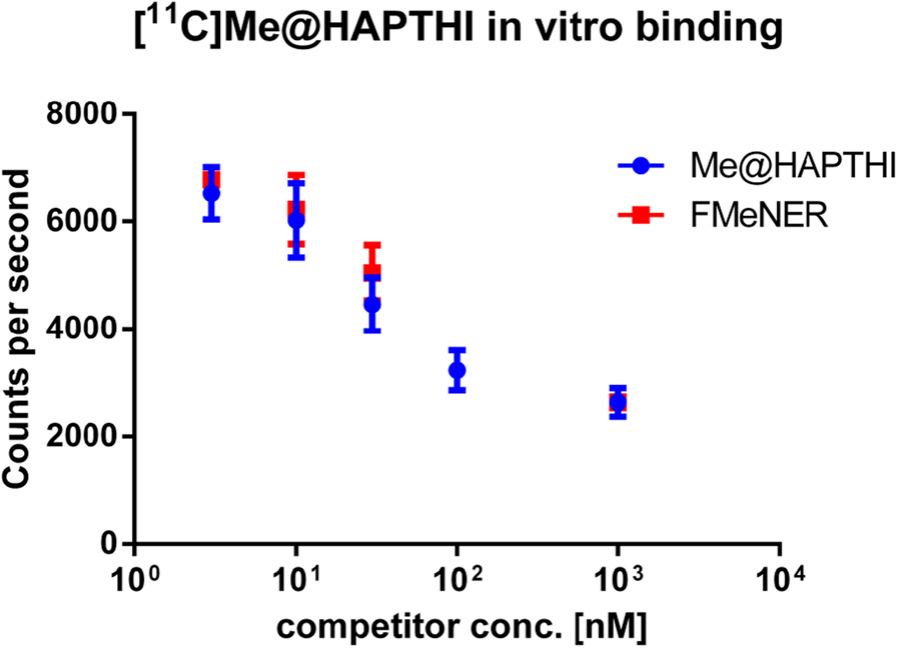
NET-binding of [^11^C]Me@HAPTHI on human NET expressing cell membranes using a harvesting protocol. Competition was done using different concentrations of Me@HAPTHI and FMeNER-D2 (1, 3, 10, 30, 100 and 1000 nM)

Moreover, harvesting experiments with [^11^C]Me@HAPTHI using hNET expressing membranes were performed according to the affinity testing protocol. Thereby, a concentration-dependent displacement of [^11^C]Me@HAPTHI was observed for all tested competitor substances (cold FMeNER-D2 or Me@HAPTHI), and the counts were corrected for decay (Fig. 8). Using Graph Pad Prism, data correlation revealed akin-binding displacement behaviour for both cold Me@HAPTHI as well as the established NET ligand FMeNER-D2 (*n* ≥ 3 triplicates).

## Discussion

[^11^C]Me@HAPTHI presents a large stride towards an improved, novel, conveniently producible PET tracer for NET imaging. This study comprises the first radiochemical preparation, quality control and in vitro evaluation of this novel candidate PET-tracer. We describe its affinity, selectivity, lipophilicity and its potential to penetrate the BBB as well as metabolic stability. Moreover, the in vitro binding behaviour of [^11^C]Me@HAPTHI to human NET cell membranes as well as human and rodent tissue slices was examined.

The excellent affinity of Me@HAPTHI (*K*_d_ hNET = 0.21 ± 0.07 nM) and exceptional selectivity of our candidate NET PET ligand present the ideal ground for a further evaluation of this tracer. Moreover, a lower non-specific binding can be expected, as the described radioligand is less lipophilic than previous NET PET tracers based on reboxetine (logD Me@HAPTHI = 2.21, logD FMeNER-D2 = 2.73). Based on the in vitro data acquired, successful BBB penetration by [^11^C]Me@HAPTHI may be expected. This assumption is supported by immobilized artificial membrane chromatography results showing Me@HAPTHI to be within the discussed range of permeability *P*_m_ values.

Additionally, the high radiochemical yields and feasible radiosynthetic availability favour our newly developed NET radioligand. The employed ^11^C-methylation reaction can be implemented at any PET facility with a cyclotron. Hence, this study presents a large stride towards a highly affine, selective and routinely available radiotracer. Moreover, in vitro stability of [^11^C]Me@HAPTHI against human liver microsomes, containing all human liver cytochrome P450 enzymes, is excellent (99.6 ± 0.3 % intact tracer after 60 min). In contrast, other existing PET tracers show significant metabolic degradation within this timeframe (e.g. [^11^C]MeNER, [^11^C]DASB or [^11^C]WAY-100635 [15, 44, 45]). Also in the rodent setting, where highly increased turnover rates of the enzymes are present, a sufficient metabolic stability of [^11^C]Me@HAPTHI was observed (29.26 ± 1.95 % intact, 60 min).

Furthermore, a plasma free fraction of 8.4 % was determined in ultrafiltration experiments, which was in a similar range with other clinically successful PET-tracers (e.g. [^11^C]ADAM).

In vitro binding studies revealed specific displaceable binding in human brain regions and rat heart, indicating towards a promising further use of this tracer in in vivo studies. Binding displacement was observed in competition experiments with different NET ligands FMeNER-D2 and Me@HAPTHI in a concentration-dependent manner. The high radiotracer uptake areas matched with the high NET-density regions identified by immunohistochemistry. Therefore, specific NET uptake of [^11^C]Me@HAPTHI can be affirmed. While this specific NET binding may be valid on ex vivo tissue, the question of binding behaviour on a cellular level was raised. Therefore, in vitro binding studies on human NET membranes were performed in a cell harvesting protocol. In these cell-based experiments, which used the same parameters as autoradiography studies (i.e. incubation time and buffer), a comparable concentration-dependent binding displacement was found for both competitors FMeNER-D2 and Me@HAPTHI. Therefore, selective NET-uptake for our novel PET ligand [^11^C]Me@HAPTHI could be confirmed on a cellular and on a human and rat tissue level.

Thus, [^11^C]Me@HAPTHI was showing highly promising results in vitro so far and might therefore become an improved, routine NET PET tracer. As a next step, small animal experiments will be performed to further elucidate the in vivo behaviour of [^11^C]Me@HAPTHI.

## Conclusions

A number of key properties have been discussed in the presented study, indicating that the benzothiadiazole dioxide [^11^C]Me@HAPTHI presents a viable and improved NET PET tracer.

We demonstrated its outstanding affinity and selectivity, its great stability in human liver microsomes, as well as promising results from in vitro autoradiography. Therefore, these data encourage us for an in vivo application of this compound in small animal PET experiments in the future. On these grounds, [^11^C]Me@HAPTHI might improve clinical NET imaging.

## Additional file

Additional file 1:
**Supplementary data on affinity testing, metabolic stability assessments and autoradiography.** Detailed methods for synthesis of precursor and reference compounds, the affinity testing of the new radiolabelled ligand via NET-expressing membrane binding protocol, as well as detailed procedures to autoradiography, immunohistochemistry and metabolic stability testings.
