# Supplementary material for: Radiosynthesis and first preclinical evaluation of the novel norepinephrine transporter pet-ligand [11C]ME@HAPTHI
Source: EJNMMI Res. 2015 Jun 10;5:34. doi: 10.1186/s13550-015-0113-3 (PMC4467816; doi:10.1186/s13550-015-0113-3)
Supplement: Additional file 1: — Supplementary data on affinity testing, metabolic stability assessments and autoradiography. Detailed methods for synthesis of precursor and reference compounds, the affinity testing of the new radiolabelled ligand via NET-expressing membrane binding protocol, as well as detailed procedures to autoradiography, immunohistochemistry and metabolic stability testings. [file 13550_2015_113_MOESM1_ESM.doc]

**Radiosynthesis and first preclinical evaluation of the Novel Norepinephrine Transporter PET-ligand [^11^C]Me@HAPTHI**

**Supplementary Information:**

Authors:

Christina Rami-Mark^1,2^ christina.rami-mark@meduniwien.ac.at

Neydher Berroterán-Infante^1,2^ neydher.berroteran@meduniwien.ac.at

Cecile Philippe^13^ cecile.philippe@meduniwien.ac.at

Stefanie Foltin ^1^ stefanie.foltin@aon.at

Chrysoula Vraka^1^ chrysoula.vraka@meduniwien.ac.at

Alexander Hoepping^4^ a.hoepping@abx.de

Rupert Lanzenberger^5^ rupert.lanzenberger@meduniwien.ac.at

Marcus Hacker^1^ marcus.hacker@meduniwien.ac.at

Markus Mitterhauser^1,3^,*^,#^ markus.mitterhauser@meduniwien.ac.at

Wolfgang Wadsak^1,2,#^ wolfgang.wadsak@meduniwien.ac.at

*Affiliations:*

*^1^Medical University of Vienna, Department of Biomedical Imaging and Image-guided Therapy, Division of Nuclear Medicine, Vienna, Austria*

*^2^University of Vienna, Department of Inorganic Chemistry, Vienna, Austria*

*^3^University of Vienna, Faculty of Life Sciences, Department of Technology and Biopharmaceutics, Vienna, Austria.*

*^4^ABX Advanced Biochemical Compounds, Radeberg, Germany*

*^5^Department of Psychiatry and Psychotherapy, Division of Biological Psychiatry, Medical University of Vienna, Austria.*

# contributed equally

***Precursor synthesis:***

Briefly, synthesis of (2S)-4-(2,2-Dioxido-3-phenyl-2,1,3-benzothiadiazol-1(3H)-yl)-1-(methylamino)butan-2-ol followed the route described by Neill et. al.[1, 2] Briefly, *N*-phenyl-o-phenylenediamine was reacted with sulfamide to afford 1-Phenyl-1,3-dihydro-2,1,3-benzothiadiazole 2,2-dioxide in moderate yields. The benzothiadiazole 2,2-doxide was then reacted with commercially available (S)-2-(oxiran-2-yl)ethanol under Mitsunobu conditions employing azodicarboxylic dipiperidide as reagent to afford the intermediate **1** which was converted to the free amine **HAPTHI** and the methylamine **Me@HAPTHI** by ring opening with ammonia or methylamine. All intermediates and final products were purified by flash chromatography over silica gel and analytical results are in agreement with their structure and with the data given in the literature.





**Figure 1**: Synthesis of precursor and reference compounds: a) sulfamide, diglyme, reflux; b) (b) (S)-2-(oxiran-2-yl)ethanol, triphenylphosphine, ADDP, THF; (c)Me-NH_2_, EtOH, 100° C, 3 min, microwave; d) NH_4_OH, 25 % NH_3_, EtOH, rt, 12 h.

***Affinity and Selectivity testing:***

The affinity of new radiolabeled ligand was determined in a NET-expressing membrane binding protocol [3-5]. The competitive binding experiments were performed in glass test tubes, filled with 350 µL of the new ‘cold’ (=non-radioactive) reference compounds, 100 µL of the membrane suspension (in assay buffer; 3 µg protein/unit, human Norepinephrine Transporter RBHNETM400AU, Perkin Elmer) and 50 µL of a 2 nM [^3^H]-nisoxetine*HCl solution (in assay buffer, 70-87 Ci/mmol, NET1084; Perkin Elmer). For non-specific binding 10 µM reboxetine (Sigma-Aldrich, Vienna, Austria) were used; and for total binding (control) only [^3^H]-nisoxetine*HCl, buffer and membrane suspension were incubated. After incubation time, binding was quenched with ice cold buffer, and membrane bound radioactivity was recovered by rapid vacuum filtration through GF/C glass fiber filters (Whatman® Inc., Clifton, USA) presoaked in assay buffer containing 0.3% polyethylene imine (PEI). Filters were washed 3 times with 4 mL assay buffer and transferred into β-counting vials. After addition of a β-scintillation cocktail (2 mL Ultima Gold^TM^, biodegradeable, Perkin Elmer), the tubes were shaken for 20 min and then counted. Data from the competition plots (as arithmetic means of values derived from three different assays, each in triplicate for each compound) were analyzed and subsequently IC_50_ and K_i_ values were calculated using GraphPad Prism® software (San Diego, USA).

To determine the selectivity of the tested compounds towards NET in comparison to the dopamine (DAT) and serotonin transporter (SERT), respectively, assays similar to those described for NET were performed. DAT and SERT expressing membranes were used instead of NET-membranes (hSERT: 9 μg protein/unit, RBHSTM400UA; and hDAT: 12.7 µg protein/unit, RBHDATM400UA, Perkin Elmer, Waltham, USA).

IC_50_ and K_i_ values were obtained in analogy to NET experiments. Ratios DAT/NET and SERT/NET were determined.

***Metabolic stability testing:***

To investigate the metabolic fate of [^11^C]Me@HAPTHI, microsomal incubations were performed. As results, both the percentage of test compound metabolized after a certain time and the biological half-life were determined.

Briefly, each pooled human or rat liver microsomes (BD Biosciences, Woburn, USA, 20 mg/mL in sucrose) were pre-incubated for 5 min under physiological conditions (phosphate buffer, pH 7.4, 37°C) with a NADPH-generating system (solution-A: NADP^+^, Glucose-6-phosphate and magnesium-chloride in H_2_O and solution-B: Glucose-6-phosphate dehydrogenase in sodium citrate). To this microsomal solution, 10 µL of the new NET-PET tracer [^11^C]Me@HAPTHI were added and incubated at 37°C (NB: 2 % ethanol in the final incubation solution should not be exceeded, in order to avoid inhibition of various enzymes like CYPs and UDP-GA)[6-9]. Enzymatic reactions were stopped at the respective timepoints by adding one volume of an ice-cold methanol/ACN mixture (10/1 v/v). The mixtures were vortexed, followed by a centrifugation step (23.000g, 5 min, RT). Aliquots of the obtained supernatant were analysed by radio-HPLC.

***Plasma protein binding***

For the determination of free fraction in human pooled plasma an ultrafiltration protocol according to previously published methods was used [10-13].Briefly, 200µL aliquots of pooled human plasma, spiked with the PET-tracer [^11^C]Me@HAPTHI (each as triplicates), were pipetted into ultrafiltration vials (Amicon Centrifree; Millipore, Bedford, USA) and the starting activities measured in a gamma counter. After incubation for 20 min at room temperature, the Centrifree vials were centrifuged for 50 minutes at 1800g. Subsequently, an aliquot of 50 µL of the ultrafiltrate was taken and measured in the γ-counter. The plasma free fraction was subsequently calculated as the ratio of the ultrafiltrate activity to the total plasma activity. To determine the amount of unspecific binding of [^11^C]Me@HAPTHI to the filter matrix of the Centrifee, triplicate aliquots of the radiotracer in phosphate buffer (pH 7.4) were also processed in the same manner as the plasma samples. The final free fraction value was corrected for this unspecific filter binding.

***Autoradiography, Nissl Staining and Immunohistochemistry***

Human brain tissue (cortex, thalamus, hippocampus, cerebellum, and hypothalamus) was obtained deeply frozen from the Neuro-Biobank of the Medical University Vienna.
Upon cutting, all tissue was stored at ‑80 °C. Before cutting, tissue blocks were thawed slowly within 12 h to -20 °C. The organs were cut at -20°C in a micro-cryotome into 10 µm thick slices and thaw mounted onto object slides. Until the beginning of the experiment, slices were stored at -80°C.

A buffer for pre-incubation (50 mM TRIS HCl in aqua dest., pH 7.4) and a buffer for the experiment (50 mM TRIS HCl, pH 7.4, 300 mM NaCl, 5 mM KCl, 0.1%w/v ascorbic acid in aqua dest.) were prepared the day before the experiment and stored at 4 °C.

Tissue sections were allowed to thaw to RT upon thorough vanishing of condense water residues (5-10min). Subsequently, the slices were pre-incubated with the respective buffer at RT for 30 min in a Coplin jar. Slices were taken out of the jar and the buffer residues were removed. The tissue was instantaneously incubated for 60 min with the assay buffer containing the freshly prepared radioligand [^11^C]Me@HAPTHI in a concentration of 40-200 nM per tissue slice.

Non-specific binding was determined by co-incubation with excess Nisoxetine (10 µM). For competition experiments, non-radioactive FMeNER-D2, an established NET-PET tracer, and Me@HAPTHI were added to the incubation solution in different concentrations. To the naïve incubation solution (baseline), a vehicle containing acetonitrile was added in the same concentration as used for the dissolution of the competitors (i.e. 0.3% acetonitrile in the final incubation solution).

After 1 h the incubation was stopped and the slices were washed twice for 5 min in a jar with ice-cold incubation buffer. Finally, the tissue slides were washed with ice-cold water to remove all residual salts and dried under a smooth air stream. Subsequently, the dry tissue slices were placed on Phosphor-Imager films in a lead shielded cassette for an exposure time of 12 h. Following the analysis of the films with a Cyclone Phosphor Imager, regions of interest (ROI) were analyzed using OptiQuant® software. The signal obtained (digital light units/mm2=DLU/mm^2^) was proportional to the radioactivity on the film. Using a calibration curve for each experiment, DLUs were converted into kBq/mm^2^. Furthermore, for calculation of NET-density, kBq were converted into fmol by taking into account the specific activity of the radiotracer preparations. All data was exported to Microsoft Excel for statistical analysis and the percentage of total specific binding was calculated.

***Nissl Staining***

The same tissue slices were stained after autoradiography with cresyl violet [5, 14, 15] to demonstrate the Nissl substance in the neurons and cell nuclei. The fixed (10% formalin solution, neutral buffered) tissues were incubated in a cresyl violet solution (0.1 % cresyl violet, 1 mL AcOH, aqua dest. ad 100 mL). After rinsing of the sections with water, the fixed brain tissue samples were dehydrated by subsequent immersion for 2 min each in 70 %, 90 %, 95 % (+2 drops glacial acid) and 100 % ethanol baths at room temperature. Thereafter, the slices were cleared in two changes of xylene (each 5 min) and the dehydrated sections were mounted with Histofluid.

***Immunohistochemistry***

Immunohistochemical staining experiments were performed on rat and human tissue cryo-slices, vicinal to the slices used for autoradiographic experiments. The staining procedure was a modification of a general protocol as published in detail previously [5, 16]. Briefly, the tissue was fixed in Aceton/MeOH 50/50 and dried. After surrounding the tissue slices with a barrier pen, endogenous peroxidase activity was blocked with 0.3 % hydrogen peroxide (in PBS). Furthermore, endogenous Avidin/Biotin was blocked using the dedicated kit. Subsequently, the tissue was incubated with goat blocking serum to significantly reduce unspecific binding. Then, the slices were incubated with a 1:100 diluted primary NET-antibody (anti-NET-antibody (H-67), sc67216) and a rabbit primary antibody isotype control (negative isotype control) for 14 h at 4 °C in a wet chamber. After incubation with the biotinylated secondary antibody (goat-anti-rabbit IgG), an Avidin/Biotin-complex solution (Vectastain kit, reagent A: Avidin dehydrogenase, reagent B: biotinylated enzyme) was added and incubated. Subsequently, the tissue was incubated with DAB (3,3'-diaminobenzidine) solution and Mayer’s Hemalaun was used as a counterstaining. The slices were dehydrated and coverslipped using an organic mounting medium (Histofluid). Slices were stored at room temperature and evaluated by visual microscopy (reflected-light microscope (Olympus IMT-2) equipped with a camera (Olympus XC50) and imaging software, Cell B, Olympus, Germany). Brightness and contrast were adjusted with Cell B software.

***References:***
